# Primary intestinal type mucinous adenocarcinoma of the ovary diagnosed during pregnancy – Maternal and neonatal treatment and outcomes: A case report

**DOI:** 10.3389/fmed.2023.1138447

**Published:** 2023-03-29

**Authors:** Eliane Azeka Hase, Henrique Norio Shinohara, João Alberto Garcia Alves Filho, José Carlos Sadalla, Alexandre Muxfeldt Ab́saber, Rossana Pulcineli Vieira Francisco

**Affiliations:** ^1^Department of Obstetrics and Gynecology, Hospital das Clínicas, Faculty of Medicine, University of São Paulo Medical School, São Paulo, Brazil; ^2^Graduate Program, University of São Paulo Medical School, São Paulo, Brazil; ^3^Department of Gynecologic Oncology, Institute of Cancer of São Paulo, São Paulo, Brazil; ^4^Department of Pathological Anatomy, Hospital das Clínicas, Faculty of Medicine, University of São Paulo Medical School, São Paulo, Brazil

**Keywords:** cancer, pregnancy, ovary, mucinous carcinoma, management

## Abstract

The incidence of ovarian cancer during pregnancy is low. Most adnexal tumors removed during pregnancy are benign, with ovarian carcinomas found in approximately 1: 10,000–1: 50,000 pregnancies. Literature on this disease is scarce and consists mostly of retrospective studies and case reports. We report the case of a pregnant patient who presented with a primary intestinal-type mucinous adenocarcinoma of the ovary and underwent unilateral salpingo-oophorectomy, with no additional surgical or chemotherapy treatment after the histological diagnosis, despite an infiltrative stromal invasion pattern. To the best of our knowledge, no such case has been previously reported. Conservative treatment in this case of early ovarian carcinoma is possible during pregnancy and should be performed in the Department of Gynecological Oncology and Obstetrics of a tertiary referral hospital. Given the possibility of disease recurrence, such patients require strict clinical oncological surveillance, specialized prenatal care, and assistance from a multidisciplinary team to improve the maternal and perinatal outcomes.

## 1. Introduction

The incidence of ovarian cancer during pregnancy is rare, with ovarian carcinomas occurring in approximately 1: 10,000–1: 50,000 pregnancies (1, 2). Most adnexal tumors removed during pregnancy are benign. The main reason for doing a surgical approach for adnexal tumors during pregnancy is the risk of rupture, torsion, and malignancy. The best treatment for ovarian carcinoma during pregnancy has not yet been established because this condition is rare; the literature on this subject is scarce, consisting mostly of retrospective studies and case reports (3). However, there is no report of a primary intestinal-type mucinous adenocarcinoma of the ovary with a pattern of infiltrative stromal invasion concomitant with pregnancy. The lack of specific guidelines, notwithstanding.

Treatment should include attention to the stage of the disease, gestational age, and the wishes of the patient (4–8). In general, this also involves conservative surgery with unilateral adnexectomy and fetal preservation (5, 9). After conservative surgery, some authors have recommended the use of antineoplastic chemotherapy drugs during pregnancy to prevent the spread of malignant cells and to eliminate possible tumor cells. In our case, however, unilateral salpingo-oophorectomy was performed with no additional surgical or chemotherapy treatment after the histological diagnosis, despite an infiltrative stromal invasion pattern related to increased disease aggressiveness (10). The objective of this case report is to present a case of primary intestinal-type mucinous adenocarcinoma of the ovary and to share our experience with this rare disease diagnosed during pregnancy, the maternal and fetal outcomes, and the subsequent oncological follow-up.

## 2. Case report

### 2.1. Case presentation

**FIGURE 1:**
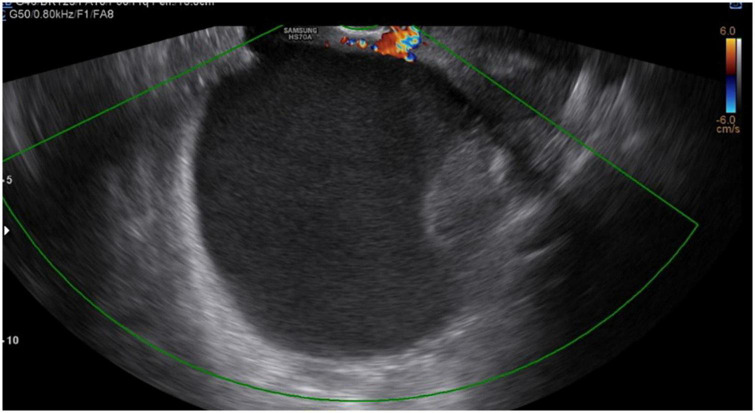
Ultrasonography (USG) image showing a cist with regular thin walls and heterogeneous, thick content, without vascular expression on Doppler.

**FIGURE 2:**
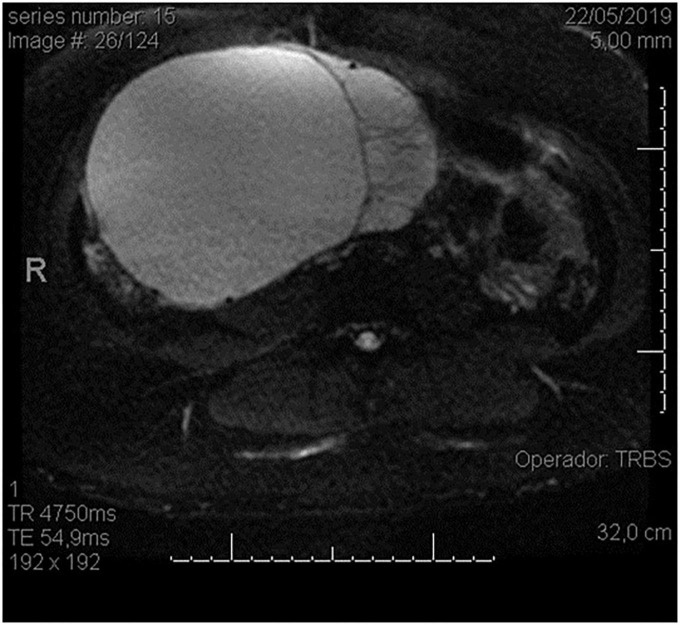
Magnetic resonance (MR) image showing a large ovarian cystic formation on the right in axial view.

**FIGURE 3:**
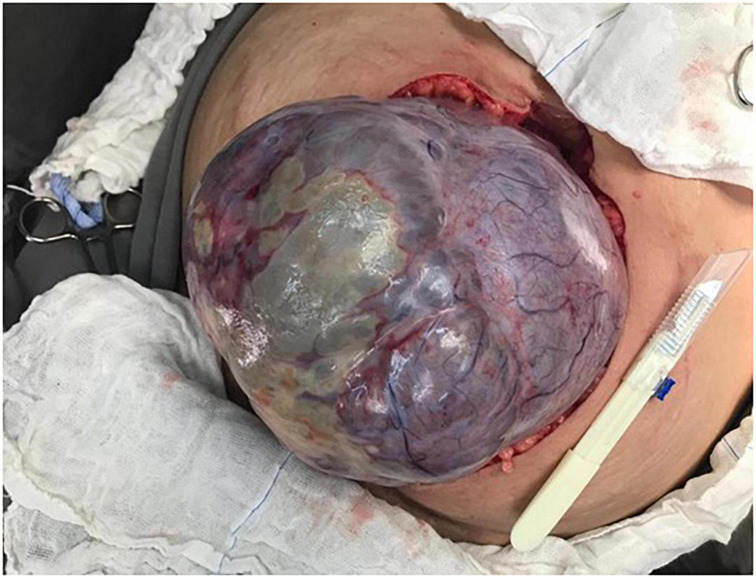
Intraoperative: macroscopic aspect of the right ovarian cystic tumor, with intact capsule, appearing through the surgical incision.

**FIGURE 4:**
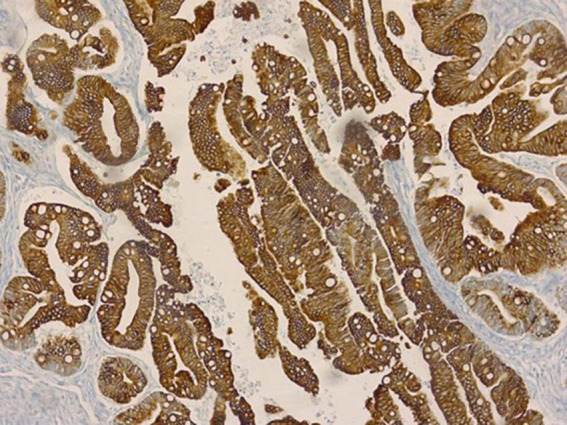
Histological patterns showing a proliferation of atypical intestinal-type glands with glandular complexes displaying an infiltrative stromal pattern.

Our patient was a 29-year-old woman, gravida 3, with a history of cesarean deliveries. During gynecological follow-up for contraception, a nodular image was identified in the right ovary on pelvic ultrasonography (USG) performed in February 2019. The cyst was solid, with regular thin walls and heterogeneous, thick content, without vascular expression on Doppler. It measured 14.5 cm×14 cm×10.4 cm (Figure 1). Due to the size of the tumor, surgical removal was indicated. However, in March 2019, during the preoperative examination, the patient was found to be pregnant and she was referred to our high-risk prenatal care. Pelvic USG was performed at that time and showed a topical pregnancy with a viable embryo at 7 weeks and 4 days, and solid-cystic formation in the right adnexal region. The serum dosage of carbohydrate antigen-125 (CA125) was 54.2 U/mL (normal, up to 35 U/mL). Additionally, magnetic resonance imaging (MRI) of the pelvis was performed on 22 May 2019 (Figure 2), confirming a voluminous, complex, multiloculated, right ovarian cystic formation with some areas of hemorrhagic content, measuring 17 cm×12 cm×18 cm (1920 cc estimated volume), without a fat component, compatible with a primary ovarian lesion. Due to the dimensions and characteristics of the tumor, an exploratory laparotomy was performed during pregnancy once the patient had consented. At 14 weeks and 5 days of pregnancy, on 5 June 2019, she underwent a right salpingo-oophorectomy (Figure 3), with no implants during revision of peritoneal cavity, and peritoneal fluid was collected for cytology without clinical and surgical complications. The pathology evaluation (AP) showed (Figure 4) an intestinal-type mucinous adenocarcinoma with an infiltrative stromal invasion pattern, moderately differentiated (G2) with an intact capsule, absence of neoplastic cells on the surface, and restriction of the tumor to the ovary. The ovary weighed 2,721 g, measured 20 cm×17 cm×12 cm. The cytology of the abdominal cavity was negative for neoplastic cells. Immunohistochemistry of the surgical specimen (06/05/2019) showed cytokeratin 7 (CK7)-, CK20-, and caudal type homeobox 2 (CDX2)–positive phenotypes. Based on these findings, considering stage IA (FIGO) of the disease and the pregnancy, after a joint evaluation with the oncology team of the Institute of Cancer of São Paulo (ICESP), an expectant approach was chosen, and the patient did not receive chemotherapy nor taken up for surgery during the pregnancy. In addition, to investigate the gastric or intestinal origin of the tumor, endoscopy and colonoscopy were performed at 18 weeks, both of which were normal. At 28 weeks of gestation, she developed a mild, pregnancy-specific, hypertensive disease that was initially controlled with diet, followed by 750 mg/day of methyldopa. The patient developed no other complications during prenatal care. At 40 weeks, elective cesarean section was performed (28 November 2019). The live newborn (NB) was a male with scores on the appearance, pulse, grimace, activity, and respiration (Apgar) scale of 8–9–10, and weighing 3,460 g. Intercurrent intraoperative uterine hypotonia was reversed with oxytocin, uterine massage, and 800 μg of misoprostol. Intraoperative evaluation of the abdominal cavity was performed during delivery with no evidence of tumor recurrence. The patient did well in the postoperative period and was discharged after 3 days. After delivery, the patient was followed up in the oncology department at ICESP with careful outpatient clinical examination and imaging tests (CT scans of the chest, abdomen, and pelvis). No evidence of disease recurrence was found.

Due to failure of the contraceptive method during outpatient follow-up, she ultimately became pregnant again 7 months after delivery. During the prenatal follow-up of this new pregnancy at our hospital, she presented with mild coronavirus disease 2019 (COVID-19) at the beginning of her pregnancy without clinical complications. On 17 March 2021, at 38 weeks and 5 days, she developed premature rupture of the membranes (PRMO), and a cesarean section with a left salpingectomy was performed. There were no implants during revision of peritoneal cavity. The newborn weighed 3,710 g, with Apgar scores of 9–10–10. She was discharged 3 days after delivery (19 March 2021) without clinical or obstetric complications during childbirth and puerperium. The patient is doing her follow up unit today, in the oncology department at ICESP with outpatient clinical examination (last on 29 August 2022), imaging test (last ultrasound at 19 August 2022) and tumor markers (CA125, CA 19.9, and CEA at 21 March 2022), and from the postoperative period of the tumor (June 2019) to the present day there was no evidence of disease recurrence.

### 2.2. Discussion

Ovarian tumors are found in approximately 1: 10,000 pregnancies, of which 3–6% are malignant (1). The incidence of ovarian epithelial carcinoma is 1: 12,000–1: 50,000 pregnancies (2). Intestinal mucinous adenocarcinoma is an uncommon type of primary ovarian tumor that accounts for approximately 2.4% of ovarian carcinomas (11). The pattern of invasion of the ovarian stroma can be classified into two groups: expansive and infiltrative invasion; however, a mixture of the two patterns is commonly found. Studies show that the infiltrative pattern occurs in approximately 44–63% of cases and is related to increased disease aggressiveness and mortality (10, 12). A key aspect of this case is that we did not find any description in the literature of a similar clinical case of primary intestinal-type mucinous adenocarcinoma of the ovary, with a pattern of infiltrative stromal invasion, concomitant with pregnancy, managed with conservative surgery. In the present report, primary intestinal-type mucinous adenocarcinoma of the ovary was diagnosed on the basis of its characteristics as a large, multilocular, and unilateral tumor (10, 13, 14), and on the histological and immunohistochemical study of the surgical specimen, positive for CK7, CK20, and CDX2 markers, which indicated the primary location of the tumor (10, 13, 15, 16). CA125 levels are used as a marker for the diagnosis and immunohistochemical control of primary ovarian carcinoma and have a higher positivity rate in serous carcinomas and a lower frequency in mucinous carcinomas. In our case, the CA125 levels were raised; however, blood markers could be physiologically elevated during pregnancy (3, 10, 14). The objective of this case report was to share our experience with a rare disease diagnosed during pregnancy, the maternal and fetal outcomes in this case, and the subsequent oncological follow-up. This was concomitant with a new pregnancy, with a short postpartum interval, and patient survival. Ovarian epithelial cancer occurs in approximately 7% of women under 40 years old and is more frequent in postmenopausal women. In this case, the pregnant woman was 30 years old when she was diagnosed with a tumor. Although approximately 75% of women with this type of cancer are diagnosed at more advanced stages of the disease, younger patients are usually diagnosed at earlier stages and have better prognosis than older patients. Examination of the surgical specimen showed that despite its large size (20 cm) and infiltrative growth pattern, the tumor was restricted to the ovary, with an intact and moderately differentiated capsule, in line with findings in the literature. According to these reports, unlike serous carcinomas, mucinous ovarian carcinomas present mostly as early-stage tumors and tend to have a good prognosis. One explanation for indolent behavior is that most well-differentiated mucinous ovarian carcinomas have a borderline tumor component with an exclusively expansive pattern of invasion, without stromal destruction by invasion (11, 13). However, in cases with an infiltrative stromal invasion pattern, stromal destruction occurs, and the tumor is considered more aggressive and is related to more advanced stages of the disease and higher mortality. Patients with primary ovarian mucinous adenocarcinoma have a 90% five-year survival rate (14). The specific rate of intestinal-type mucinous adenocarcinomas has not been described in the literature. The main reason for deciding on a surgical approach in pregnant women with an adnexal mass is the risk of rupture, torsion, and malignancy (1). In our case, a surgical approach was chosen because of the tumor size and aforementioned indications. We performed laparotomy over laparoscopy due to the tumor size and to prioritize the removal of the mass without rupture of the cist, as it may worsen the staging and prognosis of the disease (17, 18). As our initial suspicion was not ovarian carcinoma, a unilateral salpingo-oophorectomy was performed in view of the giant adnexal tumor (>10 cm), difficulty in preserving the ovary and adnexa, and the need to maintain pregnancy. After conservative surgery for the diagnosis of carcinoma, some authors have recommended using antineoplastic chemotherapy drugs during pregnancy to prevent the spread of malignant cells and eliminate possible tumor cells (9). After the result of the pathology examination, and due to the patient’s desire to maintain pregnancy, a conservative approach was adopted, without preponing the delivery and preservation of the uterus. No additional surgical and chemotherapy after the histological diagnosis, despite an infiltrative stromal invasion pattern related to increased disease aggressiveness, was performed during pregnancy or after delivery. Due to failure of the contraceptive method during outpatient follow-up, she ultimately became pregnant again 7 months after delivery. Prenatal follow-up of this new pregnancy and cesarean delivery with left salpingectomy performed in our department showed no signs of disease recurrence or clinical or obstetric complications during delivery or puerperium. From diagnosis (June 2019) to the present day, no evidence of disease recurrence has emerged. This case illustrates the importance of adequate treatment even during pregnancy. Primary intestinal-type mucinous adenocarcinoma of the ovary is a rare disease diagnosed during pregnancy and this is a strength and a limitation of our case report as there are no certain guidelines for the type of intervention, and there is no case report similar to ours to compare the results.

When a large abdominal tumor is identified, it should be managed adequately even during pregnancy. Treatment should not be deferred until after delivery because the tumor may be malignant, and a delay may result in disease progression and consequent worsened maternal prognosis. Conservative treatment in cases of early ovarian carcinoma is possible during pregnancy, but should be performed in the Department of Gynecological Oncology and Obstetrics of a tertiary referral hospital because such a carcinoma requires rigorous clinical oncological surveillance, focused on identifying disease recurrence, and utilizing specialized prenatal care with a multidisciplinary team to improve maternal and perinatal outcomes.

## Data availability statement

The original contributions presented in this study are included in the article/supplementary material, further inquiries can be directed to the corresponding author.

## Ethics statement

The studies involving human participants were reviewed and approved by the Comissão de Ética para Análise de Projetos de Pesquisa do HCFMUSP. The patients/participants provided their written informed consent to participate in this study. Written informed consent was obtained from the participant/patient(s) for the publication of this case report.

## Author contributions

EH was the principal investigator, who provided substantial contribution to the elaboration of this work and was responsible for data acquisition, analysis and interpretation, manuscript draft, and critical revision of the manuscript for important intellectual content. HS was responsible for data acquisition and manuscript draft. JA was responsible for manuscript draft and critical revision of the manuscript for important intellectual content. JS was responsible for data acquisition and critical revision of the manuscript for important intellectual content. AA was responsible for manuscript draft, pathological anatomy study, and critical revision of the manuscript for important intellectual content. RF was responsible for critical revision of the manuscript for important intellectual content. All authors contributed to the article and approved the submitted version.

## References

[1] Mancari R Tomasi-Cont N Sarno MA Azim HA Jr. Franchi D Carinelli S Treatment options for pregnant women with ovarian tumors. *Int J Gynecol Cancer.* (2014) 24:967–72. 10.1097/IGC.0000000000000161 24978707

[2] Palmer J Vatish M Tidy J. Epithelial ovarian cancer in pregnancy: a review of the literature. *BJOG.* (2009) 116:480–91. 10.1111/j.1471-0528.2008.02089.x 19250360

[3] Xu T Wang L Jia Y Jia Z Li Z Cui S Long-term multidisciplinary integrative therapy management resulted in favorable outcomes for ovarian cancer during pregnancy: a case report and literature review. *J Ovarian Res.* (2019) 12:108. 10.1186/s13048-019-0584-3 31711529 PMC6844043

[4] Grigoriadis C Eleftheriades M Panoskaltsis T Bacanu AM Vitoratos N Kondi-Pafiti A Ovarian cancer diagnosed during pregnancy: clinicopathological characteristics and management. *G Chir.* (2014) 35: 69–72. 24841681 PMC4321589

[5] Korenaga TK Tewari KS. Gynecologic cancer in pregnancy. *Gynecol Oncol.* (2020) 157:799–809. 10.1016/j.ygyno.2020.03.015 32268951 PMC7380448

[6] Han SN Verheecke M Vandenbroucke T Gziri MM Van Calsteren K Amant F. Management of gynecological cancers during pregnancy. *Curr Oncol Rep.* (2014) 16:415. 10.1007/s11912-014-0415-z 25344340

[7] Fruscio R de Haan J Van Calsteren K Verheecke M Mhallem M Amant F. Ovarian cancer in pregnancy. *Best Pract Res Clin Obstet Gynaecol.* (2017) 41:108–17. 10.1016/j.bpobgyn.2016.09.013 28029502

[8] Amant F Berveiller P Boere IA Cardonick E Fruscio R Fumagalli M Gynecologic cancers in pregnancy: guidelines based on a third international consensus meeting. *Ann Oncol.* (2019) 30:1601–12. 10.1093/annonc/mdz228 31435648

[9] Lavazzo C Minis EE Gkegkes ID. Current management of gynecologic cancer in pregnancy. *J Turk Ger Gynecol Assoc.* (2018) 19:104–10. 10.4274/jtgga.2018.0044 29699959 PMC5994818

[10] Hart WR. Mucinous tumors of the ovary: a review. *Int J Gynecol Pathol.* (2005) 24:4–25. 15626914

[11] Seidman JD Kurman RJ Ronnett BM. Primary and metastatic mucinous adenocarcinomas in the ovaries: incidence in routine practice with a new approach to improve intraoperative diagnosis. *Am J Surg Pathol.* (2003) 27:985–93. 10.1097/00000478-200307000-00014 12826891

[12] Chen S Leitao MM Tornos C Soslow RA. Invasion patterns in stage I endometrioid and mucinous ovarian carcinomas: a clinicopathologic analysis emphasizing favorable outcomes in carcinomas without destructive stromal invasion and the occasional malignant course of carcinomas with limited destructive stromal invasion. *Mod Pathol.* (2005) 18:903–11. 10.1038/modpathol.3800366 15696121

[13] Vang R Gown AM Barry TS Wheeler DT Yemelyanova A Seidman JD Cytokeratins 7 and 20 in primary and secondary mucinous tumors of the ovary: analysis of coordinate immunohistochemical expression profiles and staining distribution in 179 cases. *Am J Surg Pathol.* (2006) 30:1130–9. 10.1097/01.pas.0000213281.43036.bb 16931958

[14] Babaier A Ghatage P. Mucinous cancer of the ovary: overview and current status. *Diagnostics (Basel).* (2020) 10:52. 10.3390/diagnostics10010052 31963927 PMC7168201

[15] Silverman JF Zhu B Liu Y Lin X. Distinctive immunohistochemical profile of mucinous cystic neoplasms of pancreas, ovary and lung. *Histol Histopathol.* (2009) 24:77–82. 10.14670/HH-24.77 19012247

[16] Tabrizi AD Kalloger SE Köbel M Cipollone J Roskelley CD Mehl E Primary ovarian mucinous carcinoma of intestinal type: significance of pattern of invasion and immunohistochemical expression profile in a series of 31 cases. *Int J Gynecol Pathol.* (2010) 29:99–107. 10.1097/PGP.0b013e3181bbbcc1 20173494

[17] Falcetta FS Lawrie TA Medeiros LRF Rosa MI Edelweiss MI Stein AT Laparoscopy versus laparotomy for FIGO stage I ovarian cancer. *Cochrane Database Syst Rev.* (2016) 2016:CD005344. 10.1002/14651858.CD005344.pub4 27737492 PMC6464147

[18] Haan J Verheecke M Amant F. Management of ovarian cysts and cancer in pregnancy. *Facts Views Vis Obgyn.* (2015) 7:25–31. 25897369 PMC4402440

